# Neuronal Heterotopy in a Patient with Wiedemann–Steiner Syndrome Caused by a Truncating *KMT2A* Variant: Clinical and Genetic Correlations

**DOI:** 10.3390/reports9010037

**Published:** 2026-01-26

**Authors:** Teodora Sokolova, Hristo Ivanov, Margarita Panova, Iglika Sotkova-Ivanova, Vili Stoyanova

**Affiliations:** 1 Department of Medical Genetics, Medical University of Plovdiv, 4000 Plovdiv, Bulgaria; iglika.sotkova@mu-plovdiv.bg (I.S.-I.); vili.stoyanova@mu-plovdiv.bg (V.S.); 2 Department of Pediatrics “Prof. Dr. Ivan Andreev”, Medical University of Plovdiv, 4000 Plovdiv, Bulgaria; margarita.panova@mu-plovdiv.bg

**Keywords:** Wiedemann–Steiner syndrome, *KMT2A*, epilepsy, microcephaly, neuronal heterotopy, genotype–phenotype correlation

## Abstract

**Background and clinical significance**: Wiedemann–Steiner syndrome (WSS) is a rare autosomal dominant neurodevelopmental disorder caused by heterozygous pathogenic variants in the *KMT2A* gene, which encodes a histone lysine methyltransferase essential for chromatin regulation. Affected individuals commonly present with developmental delay, intellectual disability, behavioral disturbances, short stature, characteristic facial features, and hypertrichosis, along with variable additional congenital anomalies. Emerging genotype–phenotype correlations suggest two functional classes of *KMT2A* variants: loss-of-function variants, typically associated with the classic WSS phenotype and muscular hypotonia, and non-loss-of-function variants, which more often correlate with drug-resistant epilepsy and microcephaly. No recurrent variants or clear genotype–phenotype correlations have been established outside the CXXC domain, and most pathogenic variants are private or novel, contributing to phenotypic heterogeneity. **Case presentation**: We present a case of a 14-year-old female with a pathogenic nonsense truncating variant in the *KMT2A* gene and typical features of Wiedemann–Steiner syndrome. Additionally, the patient exhibited microcephaly and structural epilepsy due to neuronal heterotopy—features that are rarely described in individuals with truncating variants in this gene and have not been reported in the two published cases of individuals with the same mutation. **Conclusions**: This case highlights atypical genotype–phenotype correlations and expands the clinical spectrum of truncating *KMT2A* variants in Wiedemann–Steiner syndrome.

## 1. Introduction and Clinical Significance

Wiedemann–Steiner syndrome (WSS) is a rare autosomal dominant neurodevelopmental disorder caused by heterozygous pathogenic variants in the *KMT2A* gene. The gene encodes a histone methyltransferase essential for early developmental gene regulation [1,2]. The exact prevalence is unknown, but nearly 250 individuals have been reported in the medical literature to date, with cases identified across diverse ethnic backgrounds and geographic regions [2]. The syndrome is likely underdiagnosed due to its variable phenotype and overlap with other neurodevelopmental syndromes and chromatinopathies [2,3,4,5].

Most cases are sporadic, resulting from de novo mutations, though familial transmission and mosaicism have been documented [1,5]. Both males and females are affected, and there is no evidence of sex predilection. The age at diagnosis varies widely, often occurring in childhood due to developmental delay, intellectual disability, or distinctive facial and hair features, but adult diagnoses have also been reported, frequently as part of family evaluations [2,3].

Wiedemann–Steiner syndrome is recognized globally, with cohorts described in Europe, Asia, North America, and the Middle East [1,3,4,5,6]. The condition is considered a major cause of syndromic intellectual disability among patients undergoing exome sequencing for unexplained developmental delay [5].

The syndrome is characterized by a constellation of features including developmental delay, intellectual disability, distinctive facial dysmorphism (thick eyebrows, long eyelashes, synophrys, hypertelorism, broad nasal bridge), hypertrichosis, short stature, and systemic anomalies. The clinical spectrum is notably variable, with recent large cohort studies reporting additional findings such as microcephaly, seizures, and structural brain abnormalities [2,3,4].

Microcephaly is present in approximately one third of individuals with WSS, and abnormal brain imaging—including neuronal heterotopy—has been documented in nearly half of cases, most commonly involving corpus callosum anomalies or abnormal myelination [2]. Neuronal heterotopy, while not universally described, aligns with the recognized spectrum of neurodevelopmental and epileptogenic manifestations in WSS, including developmental and epileptic encephalopathies [7]. The presence of both microcephaly and neuronal heterotopy in a patient with typical WSS features further expands the phenotypic boundaries of the syndrome and supports the need for comprehensive neuroimaging and molecular evaluation in affected individuals [1,2,3].

## 2. Case Presentation

A 14-year-old girl, adopted at 4 years and 6 months of age, was evaluated for recurrent paroxysmal episodes of altered behavior accompanied by somatic symptoms. Perinatal and biological family history were unavailable. During her early childhood she was evaluated for frequent upper respiratory tract infections and a single episode of pneumonia. She had undergone adenotonsillectomy for upper airway obstruction and had no known drug or food allergies.

At 9 years of age, she was referred to an endocrinologist due to the presence of hypertrichosis. Laboratory testing revealed adrenal androgen concentrations at the upper limit of normal, with normal testosterone and 17-hydroxyprogesterone levels. Ultrasonography of the adrenal glands and ovaries was unremarkable. She received a 3-month course of spironolactone. Menarche occurred at 11 years and 6 months. Follow-up endocrinological assessment at 13 years demonstrated normal hormone levels on day 7 of the menstrual cycle, including low testosterone, and a normal thyroid ultrasound.

The patient has an established learning disability and substantial difficulties with social adaptation and relationships with her classmates. Over the preceding year, she developed episodic aggressive behavior, predominantly directed toward her mother during periods of frustration or refusal. She was evaluated by a psychiatrist and treated with aripiprazole; however, the medication was discontinued due to emotional blunting and significant weight gain.

In the year prior to her first admission to the pediatric clinic, at the age of 14, the patient developed recurrent somatic episodes characterized by headache, dyspnea, nausea, vomiting, and transient elevations in blood pressure and heart rate. These episodes typically resolved spontaneously within minutes and were observed almost daily. During events, she became unresponsive and exhibited hyperventilation.

**Figure 1:**
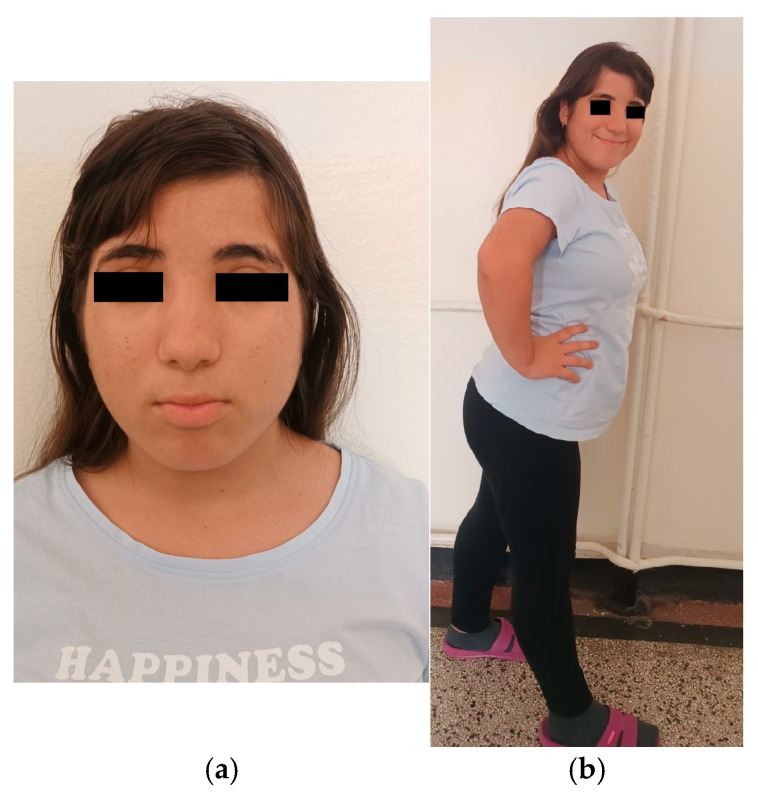
Phenotype of the patient: (**a**) Characteristic facial dysmorphism; (**b**) Brachydactyly and hypertrichosis.

On admission, her general condition was satisfactory. Anthropometric measurements were height 142.2 cm (<3rd percentile), weight 54 kg, and BMI 26.7 kg/m^2^ (85th–97th percentiles). Head circumference measured 50 cm, consistent with microcephaly; abdominal circumference measured 77 cm (90th percentile). Craniofacial dysmorphic features included reduced bitemporal diameter, hypertelorism, long eyelashes, downward-slanting palpebral fissures, mild bilateral ptosis, strabismus, broad nasal bridge and short philtrum, low-set ears and brachydactyly. Hypertrichosis was observed over the elbow joints, upper lip, and sideburn regions, legs and lower back (Figure 1a,b). Pubertal development was complete. Cardiopulmonary and abdominal examinations were unremarkable, and neurological examination revealed no focal deficits.

During hospitalization in the pediatric neurology department, two EEG studies were performed. The first recording showed no epileptiform activity, whereas the second demonstrated focal epileptiform discharges arising from the left hemisphere and a subclinical seizure, which led to the initiation of antiepileptic therapy with carbamazepine.

Cranial CT imaging was normal. Laboratory evaluation, including thyroid and sex hormone profiles, showed no abnormalities.

Psychological and psychiatric evaluation demonstrated disturbances in social interaction, with frequent mood fluctuations characterized by verbal and physical aggression directed toward her adoptive mother, as well as hyperthymic behavior toward medical staff. The patient presented with poor academic performance and the cognitive assessment revealed an IQ of 68, consistent with mild intellectual disability.

Given the combination of dysmorphic features, microcephaly, neurodevelopmental difficulties, and epilepsy, the patient was referred for medical genetic counseling. Whole-exome sequencing revealed a heterozygous truncating variant in *KMT2A*, c.3241C > T (p.Arg1081*), classified as pathogenic.

After discharge, the patient continued to experience similar paroxysmal episodes during wakefulness and sleep. Most were considered psychogenic; however, several nocturnal events remained concerning for possible epileptic origin. She was readmitted for further neurological evaluation. Follow-up EEG monitoring demonstrated persistent bitemporal epileptiform discharges despite ongoing antiepileptic treatment. Valproate was added to carbamazepine but later substituted with levetiracetam because of weight gain (6 kg for 2 months). Pregabalin was introduced for psychogenic episodes, with subsequent clinical improvement.

**Figure 2:**
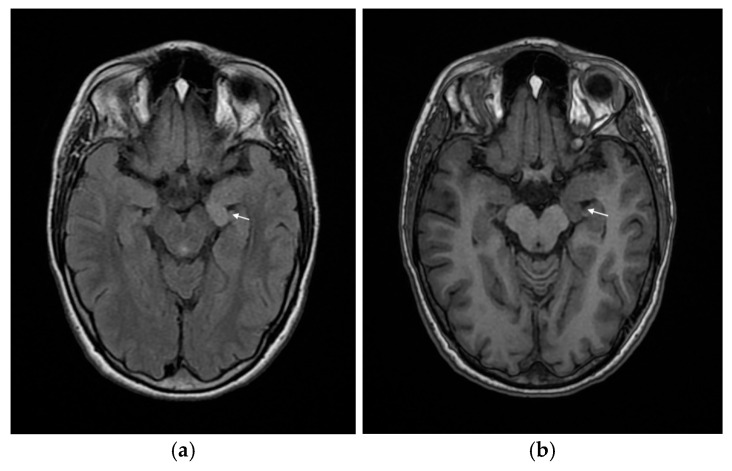
MRI of the CNS of the patient, showing neuronal heterotopy of the left hippocampus: (**a**) axial plane, FLAIR (small white arrow points the finding) (**b**) axial plane, T1 (small white arrow points the finding).

Due to the drug-resistant epilepsy and the presence of a genetic syndrome, an MRI of the central nervous system was performed, revealing neuronal heterotopy in the left hippocampal region (Figure 2a,b).

At the most recent evaluation in January 2026, the patient continued to exhibit paroxysmal events that were more suggestive of a psychogenic origin, while EEG findings showed no epileptiform activity. Levetiracetam was discontinued from the antiepileptic regimen due to electrophysiological remission. The patient was referred for psychiatric evaluation because of the suspected psychogenic origin of the episodes, frequent aggression toward the adoptive mother, autoaggressive behavior, and newly reported anamnestic data suggestive of auditory hallucinations. Quetiapine was added to the ongoing pregabalin therapy, and the patient is planned for admission to a psychiatric clinic for further evaluation.

## 3. Discussion

WSS is caused by heterozygous pathogenic variants in the *KMT2A* gene (also known as MLL), which encodes a multi-domain histone lysine methyltransferase essential for epigenetic regulation of gene expression during development [1,2,3,4,5,6,7,8,9]. Pathogenic variants include missense, nonsense, frameshift, and splice-site mutations, as well as microdeletions encompassing *KMT2A*. Both loss-of-function and missense variants have been described, with a notable clustering of missense mutations in the CXXC zinc finger domain of *KMT2A*, which is critical for binding unmethylated CpG DNA [6,10,11].

Genotype–phenotype correlations in WSS center on the type and location of pathogenic variants in the *KMT2A* gene and their association with specific clinical features. According to published cohort studies, individuals with loss-of-function (LoF) variants in *KMT2A* are more likely to present with hypotonia, while those with non-loss-of-function variants (e.g., missense) are more likely to have seizures [2]. Furthermore, missense variants in the CXXC DNA-binding domain (amino acids 1147–1195) are associated with more severe neurodevelopmental impairment, including more significant intellectual disability and developmental delay [3,6,10].

Recent cohort studies confirm these findings and expand the spectrum: LoF variants are most frequently associated with classic features such as developmental delay, intellectual disability, short stature, and hypertrichosis cubiti, while non-LoF variants may present with additional neurological manifestations, such as epilepsy [2,3]. The CXXC domain is a hotspot for pathogenic missense variants, and disruption here is mechanistically linked to altered chromatin binding and more severe neurocognitive phenotypes [6,10].

No recurrent variants or clear genotype–phenotype correlations have been established outside the CXXC domain, and most pathogenic variants are private or novel, contributing to phenotypic heterogeneity [3,4,6]. Ethnic background does not appear to significantly alter the core genotype–phenotype relationships, though some variability in feature frequency is reported [3,8].

The *KMT2A* gene mutation c.3241C > T, p.(Arg1081), found in our patient, is a heterozygous nonsense (truncating) variant that introduces a premature stop codon at amino acid position 1081, resulting in early termination of the *KMT2A* protein. This type of mutation leads to loss of function either through nonsense-mediated mRNA decay or production of a truncated, nonfunctional protein, and is a well-established pathogenic mechanism in Wiedemann–Steiner syndrome [12,13]. This mechanism is supported by both clinical and molecular studies, including direct functional analyses in patient-derived cells and review of variant spectra [2,6,13].

Genetically, truncating mutations such as p.(Arg1081*) are typically de novo and distributed throughout the gene, upstream of the FYRC domain, consistent with the location of other pathogenic variants in *KMT2A* associated with Wiedemann–Steiner syndrome. These mutations result in haploinsufficiency of *KMT2A*, meaning that a single functional allele does not produce sufficient protein for normal developmental processes [12,13]. While most published functional modeling has focused on missense variants in the CXXC domain, the pathogenic mechanism for truncating variants is consistently described as haploinsufficiency, with supporting evidence from both cellular and animal models [6,13]. There are no published studies specifically modeling the p.Arg1081* variant, but variants in similar locations and of similar type have been functionally characterized and shown to result in loss of function.

Molecularly, *KMT2A* encodes a histone methyltransferase responsible for methylation of histone H3 at lysine 4 (H3K4), a key epigenetic mark for transcriptional activation. Loss of *KMT2A* function disrupts chromatin remodeling and transcriptional regulation, particularly during early development, leading to the multisystem phenotype of Wiedemann–Steiner syndrome [13].

Transcriptomic studies in WSS consistently show that dysregulated genes tend to cluster in pathways related to neurodevelopment, chromatin organization, cell cycle control, and hair follicle biology. RNA sequencing of patient-derived fibroblasts carrying loss-of-function variants in *KMT2A* has identified altered expression of genes involved in neuronal differentiation, synaptic function, and Wnt signaling, as well as genes linked to hair growth and keratinocyte differentiation [13,14]. Notably, these changes do not point to a single dominant pathway. Instead, patterns of gene dysregulation vary across studies, reflecting the broad, pleiotropic effects of *KMT2A* haploinsufficiency. Although loss of *KMT2A* results in widespread alterations in histone methylation marks (H3K4me1 and H3K4me3), the overall impact on gene expression appears relatively modest, with most affected genes showing subtle shifts rather than pronounced up- or downregulation [10,14]. Taken together, these findings suggest that WSS pathogenesis arises from the cumulative disruption of multiple developmental and epigenetic processes, rather than from dysfunction of a single gene or pathway.

The *KMT2A* gene mutation c.3241C > T, p.(Arg1081) has been reported two times in the medical literature and has also been submitted 5 times in the ClinVar database [15]. In the published literature, this variant was first described as a de novo change in a Chinese patient diagnosed with WSS. The proband was an 8-year-old girl who exhibited several characteristic craniofacial features, including hypertelorism, a broad and flattened nasal bridge, thick eyebrows, and long eyelashes. Hypertrichosis of the elbows and lower back was also noted. Additional findings included brachydactyly, clinodactyly, normal stature, and advanced bone age. Notably, she did not present with the intellectual disability or behavioral disturbances typically associated with WSS [6].

More recently, the same variant was reported in another female patient presenting with muscle hypotonia, severe global developmental delay, and velopharyngeal insufficiency. She demonstrated several dysmorphic features compatible with WSS—such as hypertelorism, down slanting palpebral fissures, a broad nasal bridge, and brachydactyly—although she lacked the more classic traits of hypertrichosis cubiti, long eyelashes, and thick eyebrows. Macrocephaly, a feature only rarely associated with WSS, was present. In addition to intellectual disability, she exhibited behavioral disturbances including attention-deficit/hyperactivity disorder, self-injurious behavior, and hetero aggression. She developed seizures at 23 years of age; however, no information regarding associated brain malformations or neuroimaging findings was provided [16].

Our patient exhibits several dysmorphic features characteristic of Wiedemann–Steiner syndrome, including hypertelorism, a broad nasal bridge, down slanting palpebral fissures, long eyelashes, thick eyebrows, and hypertrichosis of the elbows, lower back, and lower legs, as well as brachydactyly and short stature (Figure 1a,b). She also demonstrates mild intellectual disability, episodic somatic complaints, difficulties with social interaction, impaired concentration, frequent mood fluctuations, and episodes of aggressive behavior—findings commonly reported in individuals with WSS, and overlapping with features described in one of the previously reported patients harboring the same variant. In contrast to the typical phenotype associated with truncating *KMT2A* variants, she does not exhibit muscle hypotonia. Additionally, she presents with microcephaly and structural epilepsy secondary to neuronal heterotopy (Figure 2), features that were not described in the two previously reported cases carrying the same mutation (Table 1).

Microcephaly is reported in about one third of patients with Wiedemann–Steiner syndrome (defined as head circumference below the 5th centile for age or more than 2 standard deviations below the mean) [2]. This finding is consistent across large cohorts and clinical descriptions, including both Western and Asian populations [2]. Microcephaly is considered a variable but relatively common feature of the syndrome, and its presence should prompt consideration of Wiedemann–Steiner syndrome in the differential diagnosis of syndromic microcephaly [2,6]. However, microcephaly is present in our patient but absent in the two previously reported individuals carrying the same variant; notably, one of them exhibited macrocephaly.

Structural brain malformations have been reported in about half of patients with Wiedemann–Steiner syndrome who have undergone brain imaging [2]. The most observed abnormalities include corpus callosum dysgenesis, abnormal myelination, white matter changes, and features along the Chiari I malformation spectrum. Less frequently reported findings include periventricular nodular heterotopia, choroid plexus cysts, pituitary anomalies, cortical malformations such as bilateral frontal polymicrogyria, hypoplastic optic nerves, aqueductal stenosis, third ventricle enlargement, cerebral atrophy, vermis hypoplasia, and cerebellar atrophy [2,3,5]. These observations highlight the high heterogeneity and variable penetrance of structural brain changes in WSS. While certain types of *KMT2A* mutations have been associated with differences in neurodevelopmental severity, no specific mutation has been definitively linked to a particular brain malformation in this syndrome.

Neuronal heterotopia is rare in Wiedemann–Steiner syndrome (WSS), but it has been reported. Large cohort studies indicate that neuronal heterotopy is part of the spectrum of brain MRI abnormalities observed in WSS, although the overall frequency is low compared with other neuroimaging findings, such as corpus callosum abnormalities, abnormal myelination, and Chiari 1 malformation spectrum [2,3]. When described the cases involve periventricular nodular heterotopia, which is the most common form of neuronal heterotopy [3].

Neuronal heterotopia in the hippocampal area has not been reported as a structural brain malformation in patients with Wiedemann–Steiner syndrome [1,2,3,4,5,6,7,8,9,10,11,12,13,14,15]. The medical literature does not document hippocampal-specific malformations as part of the recognized neuroimaging spectrum in Wiedemann–Steiner syndrome [2,3,5,6]. While some studies of malformations of neuronal development in other syndromes have described hippocampal abnormalities, these findings have not been associated with Wiedemann–Steiner syndrome in published cohorts or guidelines [2,5]. While some literature suggests that disruption in neurogenesis of the hippocampal formation may contribute to cognitive phenotypes in WSS, this is based on functional and neuropsychological data rather than direct imaging evidence of hippocampal structural abnormalities [17]. Therefore, hippocampal neuronal heterotopia is not a recognized neuroimaging feature of Wiedemann–Steiner syndrome in the current medical literature [2,17]. Hippocampal malformations are clinically significant due to their established association with developmental and epileptic encephalopathies, and their potential contribution to refractory epilepsy and cognitive impairment [7,17]. The long-term neurological outcomes in these patients also include persistent intellectual disability, deficits in nonverbal reasoning and visuospatial skills, executive dysfunction, below-average performance in working memory, and math [2,17,18,19].

Hippocampal heterotopia in a patient with Wiedemann–Steiner syndrome is associated with poor response to antiepileptic medications. The literature demonstrates that epilepsy in Wiedemann–Steiner syndrome frequently presents as developmental and epileptic encephalopathy, with a broad spectrum of seizure types and typically severe, drug-resistant courses [7]. The presence of hippocampal heterotopia further complicates pharmacologic management, as the abnormal neuronal networks and functional coupling between heterotopic and normotopic hippocampal tissue contribute to intractable seizures that are less likely to be controlled by standard antiepileptic drugs [20,21].

Neuronal heterotopia in the hippocampus disrupts both local and distant neural circuits, including hippocampal-prefrontal connectivity, which is critical for cognitive and behavioral regulation. Experimental and clinical data demonstrate that focal heterotopias can alter activity in remote regions such as the medial prefrontal cortex, leading to behavioral and psychiatric symptoms [21,22,23]. In the context of hippocampal heterotopia, the presence of neurons with neocortical properties further increases the risk of abnormal network dynamics and neuropsychiatric vulnerability, including psychosis and cognitive impairment.

Therefore, neuronal heterotopia in the hippocampal region in a patient with Wiedemann–Steiner syndrome can plausibly worsen psychiatric disturbances and contribute to the development of auditory hallucinations by disrupting hippocampal and connected brain circuits, although direct evidence in WSS is limited and further syndrome-specific research is needed.

The clinical picture of refractory epilepsy, mild intellectual disability, and psychiatric disturbances in our patient is consistent with the neurodevelopmental and behavioral comorbidities of WSS and the described structural malformation—neuronal heterotopia. Ongoing multidisciplinary management—including child neurology, psychiatry, and psychology—will be continued, with regular re-evaluation of antiseizure therapy for efficacy, adverse effects, and drug interactions.

Transition planning to adult care should prioritize continued neuropsychological and behavioral support, rationalization of antiseizure medications, and assessment of educational and social needs, as persistent cognitive and behavioral deficits are likely, and carer burden remains high. Regular cognitive and behavioral assessments are essential, and minimizing polypharmacy should be considered to reduce adverse effects and optimize quality of life.

## 4. Conclusions

The presence of neuronal heterotopy in our patient further broadens the neurodevelopmental phenotype of WSS and underscores the importance of comprehensive neuroimaging in affected individuals. This finding supports the need for multidisciplinary management and targeted surveillance for epilepsy and neurocognitive sequelae in WSS patients with structural brain abnormalities.

Documenting neuronal heterotopy in WSS contributes to the evolving understanding of genotype–phenotype correlations and highlights the necessity for ongoing research into the molecular mechanisms underlying neurodevelopmental and epileptogenic manifestations in this syndrome.

This case illustrates the practical value of whole-exome sequencing in patients with complex and difficult-to-diagnose clinical presentations, reinforcing current recommendations from the European Society of Medical Genetics and the American College of Medical Genetics to consider a whole-exome sequencing-first approach.

## Figures and Tables

**Table 1:** Comparison of the Clinical Features of Previously Reported Patients and the Present Case Carrying the *KMT2A* p.(Arg1081) Variant [6,16].

Patient ID	Patient 1 *	Patient 2 **	Patient 3 ***
Gender	F	F	F
Age at last examination	8y10mo	23y	14y
Stature	N	N	short
Microcephaly	−	−	+
Macrocephaly	−	+	−
Hypertelorism	+	+	+
Down slanted eye fissures	−	−	+
Blepharoptosis	+	−	+
Long eyelashes	+	−	+
Thick eyebrows	+	−	+
Broad nasal bridge	+	+	+
Bulbous nasal tip	+	−	−
Long philtrum	+	+	+
Micrognathia	+	+	−
Brachydactyly	+	+	+
Clinodactyly	+	−	−
Hypertrichosis cubiti	+	−	+
Hypertrichosis of the lower back	mild	−	+
Hypertrichosis of the lower legs	−	−	+
Velopharyngeal insufficiency	−	+	−
Muscle hypotonia	−	+	−
Intellectual disability	−	+	+
ADHD	−	+	+
Aggressive behavior	−	+	+
Seizures	−	+	+
Structural brain malformation	−	−	+

* Patient 1 [6]; ** Patient 2 [16]; *** Our patient.

## Data Availability

The original contributions presented in the study are included in the article, further inquiries can be directed to the corresponding authors.

## Abbreviations

The following abbreviations are used in this manuscript:

WSS	Wiedemann–Steiner syndrome
LoF	Loss of function
CT	Computer tomography
MRI	Magnetic resonance imaging
EEG	Electroencephalogram
BMI	Body mass index
CNS	Central nervous system
